# PI: An open-source software package for validation of the SEQUEST result and visualization of mass spectrum

**DOI:** 10.1186/1471-2105-12-234

**Published:** 2011-06-15

**Authors:** Yantao Qiao, Hong Zhang, Dongbo Bu, Shiwei Sun

**Affiliations:** 1 Institute of Computing Technology, Chinese Academy of Sciences, Beijing, 100190, China; 2 Zhejiang Gongshang University, Zhejiang, 310018, China

## Abstract

**Background:**

Tandem mass spectrometry (MS/MS) has emerged as the leading method for high- throughput protein identification in proteomics. Recent technological breakthroughs have dramatically increased the efficiency of MS/MS data generation. Meanwhile, sophisticated algorithms have been developed for identifying proteins from peptide MS/MS data by searching available protein sequence databases for the peptide that is most likely to have produced the observed spectrum. The popular SEQUEST algorithm relies on the cross-correlation between the experimental mass spectrum and the theoretical spectrum of a peptide. It utilizes a simplified fragmentation model that assigns a fixed and identical intensity for all major ions and fixed and lower intensity for their neutral losses. In this way, the common issues involved in predicting theoretical spectra are circumvented. In practice, however, an experimental spectrum is usually not similar to its SEQUEST -predicted theoretical one, and as a result, incorrect identifications are often generated.

**Results:**

Better understanding of peptide fragmentation is required to produce more accurate and sensitive peptide sequencing algorithms. Here, we designed the software PI of novel and exquisite algorithms that make a good use of intensity property of a spectrum.

**Conclusions:**

We designed the software PI with the novel and effective algorithms which made a good use of intensity property of the spectrum. Experiments have shown that PI was able to validate and improve the results of SEQUEST to a more satisfactory degree.

## Background

With the booming scale of spectra data, various software have been developed to identify proteins, such as SEQUEST [1], Mascot [2], SONAR [3], TANDEM [4], OMSSA [5]. Without a quantitative understanding of the spectrum generating process, the widely used database searching algorithms, such as SEQUEST and MASCOT, adopt a simple fragmentation model to predict the theoretical spectrum. For example, SEQUEST assumes that cleavage will occur at peptide bonds in a uniform manner and simply ignores the influence of neutral losses. This simple strategy tends to result in a significant deviation of the predicated spectrum from the experimental one.

In our previous studies, we designed a novel statistical model to determine some important factors that influence the global fragmentation [6] and proposed an EM method to derive the neutral loss (including ammonia loss and water loss) possibilities for amino acids [7]. We have used this model to predict theoretical spectrum. Using the derived quantitative parameters, we could generate the intensities for primary peaks (peaks corresponding to ions b and y) by simulating the tendency of cleavage towards middle, and estimate the cleave preference for a specific peptide bond and the intensities for neutral loss peaks from derived probabilities for each amino acids. Experimental results have shown that this model could predict a more realistic spectrum. In addition, we have used this prediction model to distinguish the false positive peptide identification in SEQUEST's output. For each peptide sequence reported by SEQUEST, we used our model to predict the theoretical spectrum and validate the peptide identification results according to the similarity between the theoretical spectrum and the experimental counterpart. On both LTQ and QSTAR spectra sets, this technique has helped to distinguish the false positive identification of SEQUEST.

We integrated these algorithms into an open source package PI (Peptide Identifier), which can be freely downloaded from http://www.bioinfo.org.cn/MSMS/.

## Implementation

Fragmentation of an amino acid bond in peptide produced n-terminal and c-terminal ions. In our Model, we assume the intensity of the fragmentation is related to the type of fragmentation bond and the position of this bond in the peptide. We named the model fragmentation event model. In a peptide A_1_A_2 _⋯ A_L _with L amino acids, we take P(A_i_, A_i+1_) as the effective factor for the amino acid bond type, and take f_i _as influential contribution when the bond lies in the i - th position in this peptide. So we can get an event v with a intensity α × P(A_i_, A_i+1_) × f_i_, and then, a event vector representing the fragment event of a peptide can be derived asV = v_1_, v_2_, ⋯, v_L-1_. Here, we solve a non-linear programming problem to train these parameters with a automatic built training set [6].

Meanwhile, PI also has an option to include EM algorithm method to gain the probabilities of dehydration and deamination [7]. With these probabilities, we can explain one fragmentation to multiple types of ions besides the major b and y ions, e.g.,  and their isotopic ions. Therefore, we can get a spectrum with reasonable intensity for multiple types of ions.

We have chosen the Jensen-Shannon Divergence, , as the scoring function to evaluate the similarity between the experimental spectrum and the theoretical one which is used for the final validation.

PI was written in Java and it is system independent. PI takes the results of SEQUEST as input files, reads the output format (out) files and spectrum format (dta) files, and exports a pix file in XML format. The pix file includes the scores from SEQUEST and PI and protein information. When running the program, the user should choose the input files firstly, and then specify the training set scale and the filtration condition in the process task dialog box. After this procession, PI assigns high scores to both the credible matches and those correct matches which are difficult to evaluate. Using the analysis function, PI can directly display the result pix file in curves. Moreover, the pix file also can be easily used by other software for the XML structural format.

## Results and Discussion

We used several data sets to evaluate our PI software program, e.g., Comp12vs12standSCX_LCQ, StrepPyogenes_FFE2_LTQ-FT, StrepPyogenes_OGE_LTQ, and Gygi's data [8], etc. The first three data sets are downloaded on PeptideAtlas [9], which include varieties of types of data from iontrap instruments.

Gygi's data set contains LTQ spectra data and QSTAR spectra which covers not only the spectra files, but also SEQUEST's results and Mascot's results from Gygi's lab for the convenience of an overall evaluation.

**Figure 1:**
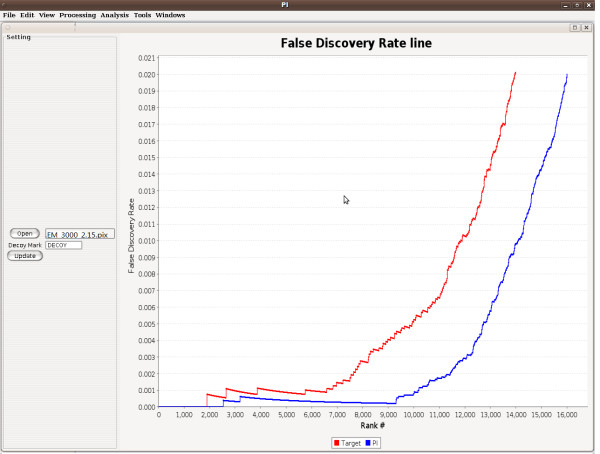
**Performance of validating the SEQUEST result on LTQ data set StrepPyogenes_OGE_LTQ**. The blue line (PI) improve the red one (SEQUEST) significantly.

The following is an example, for the data of StrepPyogenes_OGE_LTQ, in this case, there are total 169000 spectra and each dta file has correspondent out file. We set the filter thresholds as following: the ΔC_n _score was set to 0.15 and XCorr score was set to 2.0. With these thresholds, we filtered out the spectrum and peptide matches with little possibilities to be correct and focused our software on the part to include the correct matches and possible correct ones. Then, we set the training set size of 3000, and evaluated the above mentioned spectrum and peptide matches. With the pix result file, PI could give a direct illustration for comparing the results of the target software and PI self with a false positive curve shown as in Figure 1. From this figure, we can see the PI improves the SEQUEST from 9935 to 12797 at the false discovery rate 0.5% and from 11878 to 13560 at the false discovery rate 1%.

We also evaluated our PI on other data and got the similar results compared with SEQUEST (see Table 1). PI performed excellently when compared to SEQUEST on Iontrap data set. However, the result was not very stable on QTOF data set with the same setting. The reason may be that the spectra data from QTOF spectrometer have good accuracy with the m/z value, but the peaks in spectrum are of relatively low quality and have small intensity differences between the peaks. This property of spectra makes the usage of intensity lead to a less-than-satisfying result.

**Table 1:** The performance of PI and SEQUEST on different data sets, i

Data Set	PI FPR 0.5%	SEQUEST FPR 0.5%	PI FPR 1%	SEQUEST FPR 1%
Comp12vs12standSCX_LCQ	9321	7915	9546	8835

StrepPyogenes_OGE_LTQ	12877	8565	13559	10699

StrepPyogenes_FFE2_LTQFT	3820	2956	3866	3766

Gygi LTQ	10322	8431	10751	9697

Because of the intuitive and simple interface style, PI is easy and convenient to work with. A complete manual in portable document format (PDF or docx) is provided and is accessible via web pages on our web site.

**Figure 2:**
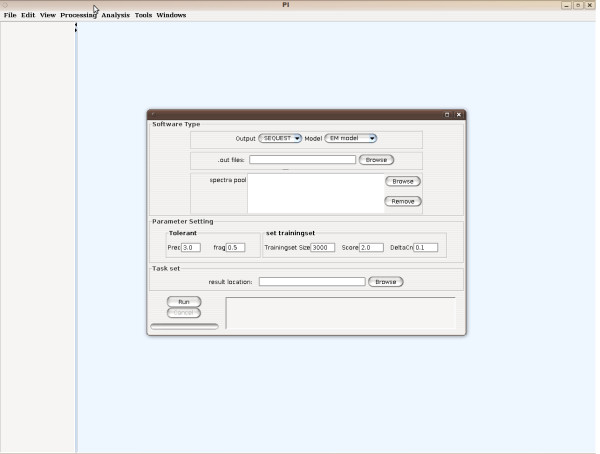
**The main panel of PI after selecting the Validation function**.

**Figure 3:**
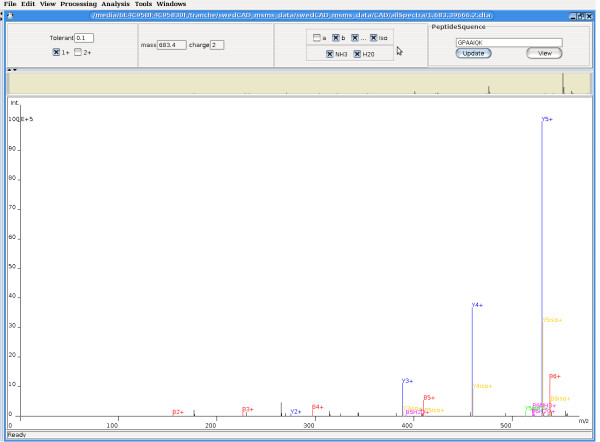
**Example of Labelling a spectrum with PI**.

The design with a GUI make it convenient for users to run the validation function based on the output files of SEQUEST and display the results. See Figure 2. Another important feature of the GUI is the capability to label the spectrum with frequent ions, such as b, y, a, dehydration ions, deamination ions and isotopic ions. This function can certainly help to analyze the match degree between the spectrum and the candidate peptide. See Figure 3.

## Conclusions

We designed the software PI with the novel and effective algorithms which made a good use of intensity property of the spectrum. Experiments showed that PI could validate the results of SEQUEST and improve the results to a satisfactory degree.

## Competing interests

The authors declare that they have no competing interests.

## Availability and requirements

Project name: PI

Project home page: http://www.bioinfo.org.cn/MSMS/

Operating system: platform independent

Programming language: Java

License: GNU

Any restrictions to use by non-academics: none

## Authors' contributions

YQ carried out the main programming work. HZ participated in design/debugging of the software. DB participated in the design of the software and performed the statistical analysis. SS conceived the software development, participated in its design and draft the manuscript. All authors read and approved the final manuscript.
